# Gaining Brain Insights by Tapping into the Black Box: Linking Structural MRI Features to Age and Cognition using Shapley-Based Interpretation Methods

**DOI:** 10.1007/s12021-025-09737-2

**Published:** 2025-10-22

**Authors:** Julia Kropiunig, Øystein Sørensen

**Affiliations:** Center for Lifespan Changes in Brain and Cognition, Department of Psychology, University of Oslo, Oslo, Norway

**Keywords:** Brain age, Cognition, Explainable AI, SAGE, Shapely values

## Abstract

Global interpretability in machine learning holds great potential for extracting meaningful insights from neuroimaging data to improve our understanding of brain function. Although various approaches exist to identify key contributing features at both local and global levels, the high dimensionality and correlations in neuroimaging data require careful selection of interpretability methods to achieve reliable global insights into brain function using machine learning. In this study, we evaluate multiple interpretability techniques such as SHAP, which relies on feature independence, as well as recent advances that account for feature dependence in the context of global interpretability, and inherently global methods such as SAGE. To demonstrate the practical application, we trained XGBoost models to predict age and fluid intelligence using neuroimaging measures from the UK Biobank dataset. By applying these interpretability methods, we found that mean intensities in subcortical regions are consistently and significantly associated with brain aging, while the prediction of fluid intelligence is driven by contributions of the hippocampus and the cerebellum, alongside established regions such as the frontal and temporal lobes. These results underscore the value of interpretable machine learning methods in understanding brain function through a data-driven approach.

**Supplementary Information:**

The online version contains supplementary material available at https://doi.org/10.1007/s12021-025-09737-2.

## Introduction

The availability of large datasets combined with an increase in computing power has led to machine learning methods becoming an increasingly important tool for analysis of neuroimaging data across various fields (Bzdok & Yeo, 2017; Bzdok et al., 2019; Davatzikos, 2019; Serra et al., 2018). In addition to diagnostics, e.g., for psychiatric disorders (Janssen et al., 2018; Nielsen et al., 2020) or detecting lesions or strokes (Nenning & Langs, 2022), researchers are getting increasingly focused on prognostic applications such as prediction of dementia risk (Pellegrini et al., 2018; Lombardi et al., 2022) and prediction of cognitive performance (Jollans et al., 2019), as well as brain age prediction (Lombardi et al., 2021; Beheshti et al., 2022; Leonardsen et al., 2022; Tanveer et al., 2023).

While machine learning models achieve high predictive accuracy by being able to detect small and complex patterns in large datasets, the mechanisms by which they function usually remain hidden and incomprehensible to the human eye. Hence, ensuring that their predictions are explainable is equally important, particularly in clinical settings.

Although interpretability and explainability are closely related concepts and more often than not used interchangeably, the machine learning literature is increasingly putting focus on their distinction. Interpretability is more concerned with presenting outputs in a human-understandable way and as a result is enabling the identification of cause-and-effect relationships in the model, whereas explainability focuses on the internal workings of the model’s decision-making (Doshi-Velez & Kim, 2017; Linardatos et al., 2020). Although strictly speaking interpretability does not include explainability and vice versa, like Linardatos et al. (2020) we consider interpretability to be the superordinate class.

Interpretability has two different aspects to it. On one hand, it is crucial to understand *why* a model predicts a specific outcome a certain way in order to establish trust in a trained model. Having insights into the grounds for decision-making is especially important for applications where the model’s output is impacting a decision that affects an individual (Rudin, 2019), and is ensured by regulations such as the General Data Protection Regulation (GDPR) in the European Union (European Commission, 2016). On the other hand, the potential for obtaining knowledge has elicited growing interest in understanding how a model behaves across whole datasets and consequently, in identifying key features driving predictions and their associated significance. Arguably, scientific progress requires opening the black box and trying to uncover the mechanisms learned by the model (Davatzikos, 2019).

The field of explainable artificial intelligence (XAI) has focused on finding solutions to these problems and has introduced methods which can generally be categorized as *local* or *global* (Samek et al., 2019; Xu et al., 2019; Lundberg et al., 2020; Covert et al., 2021). While it has been argued that models should be selected based on a trade-off between predictive accuracy and interpretability (Mateos-Pérez et al., 2018), XAI tools offer the opportunity to avoid this trade-off and enable explaining predictions made by complex and accurate models.

These tools have received limited attention in the field of neuroimaging, but recently a framework for interpreting machine learning models has been presented by Kohoutová et al. (2020). Despite the importance of both local and global interpretability, current focus predominantly lies on clinical applications, emphasizing local interpretability, where XAI methods have been employed for both brain age prediction (Lombardi et al., 2021) and for categorizing participants into healthy controls, cognitively impaired, and dementia patients (Lombardi et al., 2022; Leonardsen et al., 2023). Consequently, the exploration of global feature importance in high-dimensional machine learning models remains limited, despite its immense potential in neuroscience and health sciences in general. Traditional approaches such as regression-based methods, while interpretable, are best used for low-dimensional data (Hastie et al., 2009), i.e., single brain measures and demographics as predictors, and therefore often fail to capture complex interaction effects inherent in high-dimensional neuroimaging data.

XAI is an area of active development and exhibits notable shortcomings in applications with high-dimensional and/or highly correlated features, which are typical challenges of neuroimaging data. The following paper discusses several interpretability methods, both local and global, and demonstrates their application to neuroimaging data. In particular, we demonstrate how local interpretability methods can be aggregated to extract meaningful global insights. We provide an in-depth exploration of explainable AI tools for researchers seeking to apply machine learning methods to neuroimaging, with a particular focus on regression tasks.

The paper is structured as follows. Section Problems ofExplainable AI for Neuroimaging Data examines the limitation and challenges of XAI in the context of neuroimaging data. Section “Local Interpretability Methods” describes local model-agnostic interpretability methods, while Section “Global Interpretability Methods” covers global model-agnostic interpretability methods. In Section “Application” the presented methods are applied to two XGBoost models: the first model predicts age from segmented structural magnetic resonance imaging (MRI) data, whereas the second model predicts fluid intelligence from the same data. It is important to note that the focus here is not mainly on predicting the outcome in new observations but rather on identifying features driving the prediction with methods discussed in Sections “Local Interpretability Methods” and “Global Interpretability Methods”. Section “Discussion” provides a discussion and comparison of the advantages and disadvantages of the different methods, before we conclude in Section Conclusion. The data used cannot be shared publicly, but simulated datasets as well as R and Python code are available in the OSF archive (https://osf.io/epmgk/).

## Problems of Explainable AI for Neuroimaging Data

The problem of explaining predictions and models is as old as the problem of predicting outcomes accurately itself. As models become more complex, they usually become less interpretable. This is further exacerbated by the curse of dimensionality, which interpretability tends to suffer from, i.e., large number of features complicates model interpretation. In particular, ensemble methods such as gradient boosting (Chen & Guestrin, 2016) and random forests (Breiman, 2001) need proper tools to understand their inner workings. Simple methods for quantifying feature importance already exist for such models, e.g., partial dependence plots (PDP) and feature importance summaries (Hastie et al., 2009). These global behaviour measures tend to be straightforward and at times way too simple for clinical data due to the lack of feature independence in most real-world data. A commonly used approach consists of permuting a single feature and reevaluating the model’s performance on the modified dataset. Since the perturbation breaks the association between the response and the feature, a high decrease in performance indicates that the feature is important for accurate modelling and vice versa (Breiman, 2001). However, if features are highly correlated, the model’s performance might not drop since the information is encoded in other features, leading to potentially underestimating important features and misleading explanations (Hooker et al., 2021). Secondly, complex mechanisms in the brain are often subject to interaction effects. These modelled effects are impossible to disentangle with single feature permutations (Covert et al., 2020a).

Furthermore, results of XAI tools can be method-dependent and can yield ambiguous results depending on model and data complexity. Further complications arise from the Rashomon effect, where feature dependence causes models with comparable performance to rely on different features for their decision-making processes (Breiman, 2001). It is essential to be aware of these problems when choosing an appropriate approach for model interpretation and to potentially validate results with other explainable AI tools or different model setups. It is also advisable to be familiar with the concepts and assumptions underlying different XAI methods to facilitate an appropriate choice of tools for model interpretation. In order to properly discuss tools that circumvent the aforementioned problems in the following section we introduce the following mathematical framework.

### Setup

Consider training data $D = \{(x^i, y^i)\}_{i=1}^n$ consisting of *n* instances of an *M*-dimensional feature vector $x^i = \left( x_1^i, \dots , x_M^i \right) \in \mathbb {R}^M$ and corresponding response/label $y_{i} \in \mathbb {R}$, that has been used to train a supervised machine learning model represented as function $f: \mathbb {R}^M \longrightarrow \mathbb {R}$, so that *f*(*x*) approximates response *y* belonging to *x* as best as possible. In the following denote by $\mathcal {S}$ a subset of features in $\mathcal {M} = \{x_1,..., x_M\}$ and by $\mathcal {S}^{\mathcal {C}}$ its complement $\mathcal {M}\setminus \mathcal {S}$. Furthermore, let $x_{\mathcal {S}}$ resp. $x_{\mathcal {S}^{\mathcal {C}}}$ represent the vector containing components of *x* that are in $\mathcal {S}$ resp. $\mathcal {S}^{\mathcal {C}}$. Let $\Sigma _{\mathcal {S}\mathcal {S}^{\mathcal {C}}}$ denote the covariance between features in $\mathcal {S}$ and features in $\mathcal {S}^{\mathcal {C}}$ with $\mathcal {S}\subseteq {\mathcal {M}}$. Finally, $\textbf{x}$ represents an arbitrary but fixed instance of the feature vector.

## Local Interpretability Methods

The primary focus of local interpretability methods is to explain a prediction on an individual-level. Depending on whether the task is a classification or a regression problem, different options are available. Compared to regression models, classification models learn a well-defined decision boundary. Counterfactual explanations use this decision boundary to determine the minimal value changes needed in the observation to result in a different outcome (Wachter et al., 2017; Lucic et al., 2020). Apart from identifying pivotal features for the prediction, this approach helps determining the robustness of the prediction.

A way to tackle local interpretability for regression and classification tasks consists of (additive) feature attribution methods, i.e., the contribution of each feature adds up to the prediction of the observation. Local additive feature attribution methods have the valuable property of being extendable to global interpretability methods by averaging their absolute feature attribution values across multiple observations. One approach within this class, known as LIME, involves training a simpler, more interpretable surrogate model locally around the observation to be explained (Ribeiro et al., 2016), see also Appendix B. While LIME assumes a linear local approximation, which can provide comprehensive interpretations, explanations can become inaccurate if the model is highly nonlinear around the observation to be explained. Additionally, LIME requires sufficient data density around the observation to construct a reliable surrogate model. In contrast, Shapley values estimate each feature’s contribution to the prediction, also accounting for feature interactions (Shapley, 1953; Lundberg & Lee, 2017; Aas et al., 2021), providing more reliable explanations.

### Shapley Values

Shapley values are a concept first developed in cooperative game theory to quantify the contribution $\varphi _i$ of a single player *i* in an instance of a cooperative game (Shapley, 1953). In the context of machine learning, each feature corresponds to a player, with the aim of quantifying how much each feature contributes to the overall prediction. Shapley values are a model-agnostic approach in which the prediction $f(\textbf{x})$ for a single instance $\textbf{x}$ is explained by explaining the difference between the prediction $f(\textbf{x})$ and the global average prediction $\bar{y} = E[f(x)]$.

The Shapley value of a feature *i* is defined by

1 $$\begin{aligned} \varphi _i = \sum _{ \mathcal {S} \subseteq \mathcal {M}\setminus \{i\}} \frac{|\mathcal {S}|!(M - |\mathcal {S}| -1)!}{M!} \big ( \nu (\mathcal {S} \cup \{ i\}) - \nu (\mathcal {S})\big ),\hspace{0.3cm} i \in \{1, ..., M\}, \end{aligned}$$

where $\nu (\mathcal {S})$ is a contribution function, evaluating the contribution of the combination of features in subset $\mathcal {S}$ to the prediction. The difference $\nu (\mathcal {S} \cup \{ i\}) - \nu (\mathcal {S})$ acts as a quantifier of the contribution of feature *i* in the coalition of $\mathcal {S} \cup \{i\}$. The sum in Eq. 1 is over all possible sets of features not including feature *i*, in total $2^{M-1}$. The Shapley value for feature *i* can therefore be seen as a weighted average between the differences of the contribution *i* in every possible combination of elements in $\mathcal {M}\setminus \{i\}$. Furthermore, $\varphi _0$ is defined as the contribution of the empty set, i.e., $\varphi _0 = \nu (\emptyset )$, which coincides with *E*[*f*(*x*)] and is not attributed to any of the features.

The use of Shapley values in the context of feature contribution was first suggested by Štrumbelj and Kononenko (2010, 2014). With the notion that $\varphi _0$ represents the fixed offset as *E*[*f*(*x*)], the remaining Shapley values explain the individual contribution of the features to the prediction, i.e.,

$$\begin{aligned} f(x) = \varphi _0 + \sum _{i = 1}^{M} \varphi _i. \end{aligned}$$

Explanation methods of this form are commonly known as *additive feature attribution* methods. Every prediction is associated with a different set of Shapley values and has to be recalculated for every instance of interest. While Shapley values for the features change depending on the prediction, $\varphi _0$ does not.

It has been proven (Young, 1985) that Shapley values are the only additive feature attribution method that fulfills the following favorable properties for arbitrary $i,j \in \mathcal {M}$:

1. **Efficiency:** the sum of Shapley values over all features equals the prediction, $\varphi _0 + \sum _{i = 1}^{M} \varphi _i = \nu (\mathcal {M})$;
2. **Null effects:** if $\nu (\mathcal {S}\cup \{i\}) = \nu (\mathcal {S})$ for all $\mathcal {S} \subseteq \mathcal {M}$, then $\varphi _i = 0$;
3. **Symmetry:** if $\nu (\mathcal {S}\cup \{i\}) = \nu (\mathcal {S}\cup \{j\})$ for all $\mathcal {S}\subseteq \mathcal {M}\setminus \{i,j\}$, then $\varphi _{i} = \varphi _{j}$;
4. **Linearity:** for a combination of two prediction models *f* and *g* trained on the same features and contribution functions ν and μ, the contribution for the combined model equals the sum of the contributions of the individual models for the prediction of a specific instance, i.e., $\varphi _i(\nu + \mu ) = \varphi _i(\nu ) + \varphi _i(\mu ).$

A common approach is to compute Shapley values by solving a weighted least squares (WLS) problem (Charnes et al., 1988; Lundberg & Lee, 2017),

2 $$\begin{aligned} \min _{\varphi _0, ..., \varphi _M} \sum _{\mathcal {S} \subseteq \mathcal {M}} k(M, \mathcal {S})(\nu (\mathcal {S}) -(\varphi _0 + \sum _{j \in \mathcal {S}} \varphi _j))^2, \end{aligned}$$

where $\nu (\mathcal {S})$ corresponds to the response, $\varphi _{0}$ and $\varphi _{j}$ for $j \in \mathcal {M}$ correspond to the regression coefficients in the WLS regression, and Shapley kernel weights are given by

3 $$\begin{aligned} k(M, \mathcal {S}) = \frac{(M-1)}{\left( {\begin{array}{c}M\\ |\mathcal {S}|\end{array}}\right) |\mathcal {S}|(M - |\mathcal {S}|)}. \end{aligned}$$

In order to be able to compute the Shapley values and solve the WLS problem an appropriate function $\nu (\mathcal {S})$ is needed to describe the contribution of a specific set of features $\mathcal {S}\subseteq \mathcal {M}$. This function is supposed to replicate the prediction of $\textbf{x}$ if only values for features contained in $\mathcal {S}$ are known. A natural definition of $\nu (\mathcal {S})$ arises by conditioning the expectation on $\textbf{x}_{\mathcal {S}}$, i.e., the features pertaining to $\mathcal {S}$, yielding

4 $$\begin{aligned} \nu (\mathcal {S}) = E[f(x)| x_{\mathcal {S}} = \textbf{x}_{\mathcal {S}}] = \int f(\textbf{x}_{\mathcal {S}}, x_{\mathcal {S}^{\mathcal {C}}}) p(x_{\mathcal {S}^{\mathcal {C}}}| \textbf{x}_{\mathcal {S}}) dx_{\mathcal {S}^{\mathcal {C}}}. \end{aligned}$$

In other words, Eq. 4 computes $\nu (\mathcal {S})$ by fixing the features in $\mathcal {S}$ to their given values in $\textbf{x}$ and averaging over all values in $\mathcal {S}^{\mathcal {C}}$, weighted by their conditional probability given the features in $\mathcal {S}$.

### KernelSHAP

The conditional distribution $p(x_{\mathcal {S}^{\mathcal {C}}}| \textbf{x}_{\mathcal {S}})$ is rarely known and often hard to estimate. When features in *x* can be assumed to be independent the conditional distribution reduces to the marginal distribution function of $x_{\mathcal {S}^{\mathcal {C}}}$, $p( x_{\mathcal {S}^{\mathcal {C}}})$, and the contribution function becomes

5 $$\begin{aligned} \nu (\mathcal {S}) = \int f(\textbf{x}_{\mathcal {S}}, x_{\mathcal {S}^{\mathcal {C}}}) p(x_{\mathcal {S}^{\mathcal {C}}}) dx_{\mathcal {S}^{\mathcal {C}}}. \end{aligned}$$

Solving Eq. 2 with contribution function $\nu (\mathcal {S})$ given in Eq. 5 is known as KernelSHAP. Extensive details can be found in Aas et al. (2021). There are also computationally more efficient methods exploiting characteristics of particular models, e.g., LinearSHAP for linear models and DeepSHAP for deep learning models (Lundberg & Lee, 2017).

### Shapley Values for Dependent Features

The independence assumption underlying KernelSHAP is usually unrealistic in real-world data, and not taking the dependence structure into account can lead to erroneous results. It is therefore required to estimate the conditional dependence $p(x_{\mathcal {S}^{\mathcal {C}}}| \textbf{x}_{\mathcal {S}})$ in Eq. 4 (Aas et al., 2021).

Depending on the structure of the features, conditional expectations can be computed in different ways. In the following, mainly the case of the features adhering to a multivariate Gaussian distribution, and the case when neither the marginal distributions nor their dependence are Gaussian, hence requiring empirical estimation, will be discussed.

#### Multivariate Gaussian Distribution

Given that a multivariate Gaussian distribution with mean vector μ and covariance matrix Σ is an acceptable approximation of the distribution of the features, the conditional distribution $p(x_{\mathcal {S}^{\mathcal {C}}} | \textbf{x}_{\mathcal {S}})$ follows a multivariate Gaussian $\mathcal {N}(\mu _{\mathcal {S}^{\mathcal {C}}| \mathcal {S}}, \Sigma _{\mathcal {S}^{\mathcal {C}}| \mathcal {S}})$ with

6 $$\begin{aligned} \mu _{\mathcal {S}^{\mathcal {C}}| \mathcal {S}} = \mu _{\mathcal {S}^{\mathcal {C}}} + \Sigma _{\mathcal {S}\mathcal {S}^{\mathcal {C}}}\Sigma _{\mathcal {S}\mathcal {S}}^{-1}(\textbf{x}_{\mathcal {S}}- \mu _{\mathcal {S}}), \end{aligned}$$

and

7 $$\begin{aligned} \Sigma _{\mathcal {S}^{\mathcal {C}}| \mathcal {S}} = \Sigma _{\mathcal {S}^{\mathcal {C}}\mathcal {S}^{\mathcal {C}}} -\Sigma _{\mathcal {S}^{\mathcal {C}}\mathcal {S}}\Sigma _{\mathcal {S}\mathcal {S}}^{-1}\Sigma _{\mathcal {S}\mathcal {S}^{\mathcal {C}}}. \end{aligned}$$

Subsequently, Eq. 4 can be approximated by sampling $x^j_{\mathcal {S}^{\mathcal {C}}}$ from the conditional Gaussian distribution sufficiently many times and computing the average predicted response for fixed $\textbf{x}_{\mathcal {S}}$ and sampled $x_{\mathcal {S}^{\mathcal {C}}}$ as

8 $$\begin{aligned} \bar{\nu }(\mathcal {S}) = \frac{1}{J} \sum _{j = 1}^J f(\textbf{x}_{\mathcal {S}}, x_{\mathcal {S}^{\mathcal {C}}}^j). \end{aligned}$$

#### Empirical Conditional Distribution

In the case of the features’ marginal distribution not approximately following a Gaussian distribution, nor the features relating to each other through a Gaussian copula, relying on the aforementioned closed forms of mean and variance is not recommended and can lead to faulty results. Instead Aas et al. (2021) propose to estimate the conditional distribution in a non-parametric way that leans heavily on the idea that information on $p(x_{\mathcal {S}^{\mathcal {C}}}| x_{\mathcal {S}} = \mathbf {x_{\mathcal {S}}})$ can be inferred from samples $(x_{\mathcal {S}},x_{\mathcal {S}^{\mathcal {C}}})$ whose $x_{\mathcal {S}}$ is relatively close to $\mathbf {x_{\mathcal {S}}}$ with respect to the Mahalanobis distance

9 $$\begin{aligned} D_{\mathcal {S}}(x, \textbf{x}) = \sqrt{\frac{(x_{\mathcal {S}} - \mathbf {x_{\mathcal {S}}})^T \Sigma _{\mathcal {S}}^{-1} (x_{\mathcal {S}} - \mathbf {x_{\mathcal {S}}}) }{|\mathcal {S}|}}. \end{aligned}$$

To give more importance to training samples that are closer to $\textbf{x}$ with respect to distance function *D*, an exponential weight function $\textbf{w}_{\mathcal {S}}$ is applied to instances of the feature vector in the training set, $x^i$, i.e.,

10 $$\begin{aligned} \textbf{w}_{\mathcal {S}}(\textbf{x}, x^i) = \exp \left( -\frac{D_S(\textbf{x}, x^i)^2}{2\sigma ^2}\right) . \end{aligned}$$

The bandwidth parameter σ controls how and where the weight of the samples around $\textbf{x}$ is put. The smaller σ is, the more the weight will be distributed to a small number of samples closest to $\textbf{x}$. Conversely, a higher σ will put weight on a greater number of samples around $\textbf{x}$. In the case of highly dependent features it is advisable to use a smaller σ. Subsequently, the weights $\{\textbf{w}_{\mathcal {S}}(\textbf{x}, x^i)\}_{i = 1}^n$ can be sorted in ascending order with $x^{(j)}$ belonging to the *j*-th largest weight. The conditional expectation, i.e., the contribution function $\nu (\mathcal {S})$ in Eq. 4, can then be approximated by a weighted average of the predictions of the *J* samples that are closest to $\textbf{x}$, i.e.,

$$\begin{aligned} \bar{\nu }(\mathcal {S}) = \frac{\sum _{j = 1}^J \textbf{w}_{\mathcal {S}}(\textbf{x}, x^{(j)}) f({\textbf{x}_{\mathcal {S}}}, x_{\mathcal {S}^{\mathcal {C}}}^{(j)}) }{ \sum _{j = 1}^J\textbf{w}_{\mathcal {S}}(\textbf{x}, x^{(j)})}. \end{aligned}$$

The parameter *J* can be chosen such that the sum over *J* weights reaches a certain percentage of the total sum, or, if that should exceed a certain high number, e.g., J=5000, the parameter is set to that.

The empirical conditional distribution can also succumb to the curse of dimensionality and therefore works best if $| \mathcal {S}|$ is relatively small, e.g., $|\mathcal {S}| \le 3$. It is therefore suggested to combine both approaches with sampling from a Gaussian distribution or Gaussian Copula to compute $\bar{\nu }(\mathcal {S})$ if $|\mathcal {S}| > 3$, and sampling from the empirical conditional distribution otherwise.

### Group Shapley for High-Dimensional Feature Spaces

The number of terms in the sum in the definition of the Shapley value Eq. 1 grows exponentially with the number of features, since the number of subsets $\mathcal {S} \subseteq \mathcal {M} \setminus \{i\}$ is $\mathcal {O}(2^{M})$. Even though an approximation through sampling feature combinations $\mathcal {S}$ according to their kernel weights $k(\mathcal {M}, \mathcal {S})$, as suggested by Lundberg and Lee (2017), reduces computational complexity, the number of samples required to obtain an acceptable approximation grows quickly with *M*. Consequently, computing the Shapley values is intractable with thousands of features.

Jullum et al. (2021) suggested bypassing the problem by grouping the features into *R* groups, $G_{1}, \dots , G_{R}$, with R≤30. In this approach the $\mathcal {O}(2^{M})$ subsets $\mathcal {S} \subseteq \mathcal {M}$ are replaced by $\mathcal {O}(2^{R})$ groups $G \subseteq \mathcal {G}:= \{G_{1}, \dots , G_{R}\}$. The Shapley value for the group $G_{r}$ is now defined by

11 $$\begin{aligned} \varphi _{r} =\! \sum _{G \subseteq \mathcal {G} \setminus G_{r}} \frac{\left| G\right| ! \left( R - \left| G\right| - 1\right) !}{R!} \left( \nu \left( G \cup G_{r}\right) - \nu \left( G\right) \right) , \hspace{0.2cm} r\in \{1,\dots , R\}, \end{aligned}$$

where ν(G) now defines the contribution of all the features in group *G*.

A data-driven approach to defining groups involves grouping together the most correlated features using hierarchical clustering methods, such as dendrograms. A disadvantage of this approach may be a lack of interpretability, as the meaning of the groups is not apparent. Alternatively, in a theory based approach groups are formed based on their theoretical characteristics, requiring domain knowledge. In the context of neuroimaging, features may be grouped based on, e.g., spatial closeness or functional similarity. We show an example of this in the application section.

Finally, note that the grouping is only applied in the computation of the Shapley values. For optimizing predictive power, the machine learning models should fit on the original features.

## Global Interpretability Methods

While local interpretability focuses on explaining an individual prediction, the goal of global interpretability is to discern a feature’s importance across an entire dataset and to get insights into the overall behavior of the model. Most commonly, the problem of determining global feature importance is understood as determining the predictive power a feature holds (Covert et al., 2020b). An alternative approach involves evaluating the sensitivity of the model’s output to changes in the input (Horel et al., 2018).

While our focus is on model-agnostic methods, it is worth mentioning some commonly used model-specific techniques: feature importance quantification as number of splits on a specific feature in a random forest (Hastie et al., 2009, Ch. 15), magnitude of standardized regression coefficients in linear regression techniques such as lasso (Tibshirani, 1996) or ridge.

Global model-agnostic methods can be divided into several main categories. One notable category includes permutation and conditional permutation feature importance methods (Strobl et al., 2008; Fisher et al., 2019; Chamma et al., 2023). Another category focuses on removal-based feature importance, wherein a feature or a group of features is removed by either setting these features to default values or marginalizing them out (Covert et al., 2020a). Global variants of the Shapley values such as Mean Absolute SHAP and SAGE (Covert et al., 2020b) rely on this concept. While methods such as Mean Absolute SHAP try to raise Shapley values to a global level by averaging local Shapley values, SAGE establishes a direct connection between global interpretability and local Shapley values by explaining the mean loss (Covert et al., 2020b).

### SAGE - Shapley Additive Global Importance

Methods that measure feature importance by removing features typically underestimate the importance of features that are correlated. Conversely, methods that measure feature importance by including features underestimate the importance of complementary features, i.e., features that have more predictive power when used together. SAGE, a global additive importance measure method that represents feature importance while accounting for feature interactions in the model function, intends to remedy these drawbacks. In addition, SAGE can yield faster results than averaging over local SHAP values and offers uncertainty quantification. In general, two types of predictive power can be distinguished; universal predictive power and model-based predictive power with the latter being an approximation of the universal one. A natural definition of a measure for predictive power of a subset $\mathcal {S}$ arises by quantifying the improvement of the model’s accuracy on inclusion of the features in $\mathcal {S}$ with respect to the expected loss,

12 $$\begin{aligned} \nu _f(\mathcal {S}) = E[l(\bar{y},y)] - E[l(E[f(x_{\mathcal {S}}, x_{\mathcal {S}^{\mathcal {C}}}) | x_{\mathcal {S}}], y)], \end{aligned}$$

where l(·) is the loss function of the model, $\bar{y}$ is the average response value, and *y* is the actual response. Thus, $E[l(\bar{y}, y)]$ is the expected loss for a model with no features and $E[l(E[f(x_{\mathcal {S}}, x_{\mathcal {S}^{\mathcal {C}}}) | x_{\mathcal {S}}], y)]$ is the expected loss conditional on observing the feature values $x_{\mathcal {S}}$.

Computing SAGE values $\Phi _1,..., \Phi _M$, where $\sum _{i \in \mathcal {S}} \Phi _i$ for $\mathcal {S}\subseteq \mathcal {M}$ represents the predictive power of subset $\mathcal {S}$, can similarly to local Shapley values (Štrumbelj & Kononenko, 2014; Lundberg & Lee, 2017) be seen as a weighted least squares problem of the form

13 $$\begin{aligned} \min _{\Phi _1, ..., \Phi _M} \sum _{\mathcal {S} \subseteq \mathcal {M}} k(M, \mathcal {S})(\nu _f(\mathcal {S}) - \sum _{j \in \mathcal {S}} \Phi _j)^2, \end{aligned}$$

with kernel weights given in Eq. 3 and contribution function $\nu _f(\mathcal {S})$ defined in Eq. 12.

To avoid exponential computational costs related to the number of subsets of the features, Covert et al. (2020b) proposes several estimation approaches, including feature permutation sampling and a KernelSHAP based approach (Lundberg & Lee, 2017; Covert & Lee, 2021). The permutation based approach is characterized by sampling a permutation of the features, which are successively added to a growing subset of features $\mathcal {S}$. For every inclusion of a feature *j* to subset $\mathcal {S}$, a number of Monte Carlo samples $x_{\mathcal {S}^\mathcal {C}}^k$ is generated to evaluate how the average prediction $f(x_{\mathcal {S}}, x_{\mathcal {S}^\mathcal {C}}^k)$ reduces the loss and is attributed to feature *j*. In contrast, the KernelSHAP approach solves for unbiased SAGE values through a linear regression approximation.

## Application

### Modelling

In the following, we will demonstrate the above-mentioned techniques on a data set extracted from the UK Biobank (https://www.ukbiobank.ac.uk/) consisting of 39,625 participants (mean age = 54.74 year, standard deviation = 7.49 years) having both a neuropsychological test score for fluid intelligence (data field 20016) and a T1-weighted brain MRI scan. All scans were acquired on a 3T Siemens Skyra scanner equipped with a standard 32-channel head coil running VD13A. T1-weighted images were collected using a 3D MPRAGE sequence with 1.0 mm isotropic resolution (TR = 2000 ms, TI = 880ms). Preprocessing followed the UK Biobank imaging pipeline (Alfaro-Almagro et al., 2018), which includes gradient distortion correction, field-of-view cropping, brain extraction (FSL BET), bias field correction, tissue-type segmentation (FSL FAST), and nonlinear registration to MNI152 space (FSL FNIRT). MRI variables of interest were extracted with Freesurfer v6.0 (Dale et al., 1999; Fischl et al., 2002) and include measures for volume (V) and mean intensity (MI) of subcortical segmentations as well as cortical measurements of mean thickness (MTh) and area (A) using the Desikan-Killiany-Tourville atlas (Desikan et al., 2006; Klein & Tourville, 2012). Compound measures, such as total estimated grey volume, as well as extracerebral structures, except for the ventricles, were excluded, resulting in 179 features total. The results were obtained using Python v3.10.4 and R v4.2.1 (R Core Team, 2024). Essential packages and their specific version for Python were ’XGBoost’ v2.0.3, ’sage-importance’ v0.0.5 and ’shap’ v0.42.1, while ’shapr’ v0.2.3 (Sellereite & Jullum, 2019) was a relevant package in R.

As a central component of the modeling process, XGBoost utilizes gradient boosting, a widely used machine learning technique for classification and regression tasks. Gradient boosting combines multiple sequentially computed weak learners, each trying to minimize a loss function to further improve the predictive model (Freund, 1999; Friedman, 2001). For gradient tree boosting methods these weak learners are single trees. Among various tree boosting methods such as XGBoost (Chen & Guestrin, 2016), LightGBM (Ke et al., 2017), AdaBoost (Freund, 1999), CatBoost (Prokhorenkova et al., 2017), XGBoost has proven to be high-performing and highly robust. Gradient tree boosting is a highly efficient approach for modelling tabular data. While deep learning models have achieved great success in problems involving images, text, and audio, tree ensemble methods often outperform deep learning when applied to structured, lower-dimensional tabular data, such as MRI-derived neuroimaging inputs, which are more informative than raw images in this context (Shwartz-Ziv & Armon, 2022). We briefly review XGBoost’s formulation in Appendix A and refer to Chen and Guestrin (2016) for extensive details.

We trained two gradient tree boosting models on the brain measures: the first to predict age and the second to predict fluid intelligence. In the age model, we regressed out the effects of sex and estimated total intracranial volume (ICV) prior to training the XGBoost model. That is, input features to the model were residuals of a linear model having sex and ICV as predictors. Initially, we additionally regressed out age for the brain measures, and sex and age for the response in the fluid intelligence model, but decided to run the model without any residualizing. This is due to a potential introduction of small ICV effects on features without a statistical association with ICV. As a result, irrelevant features may become important due to the correlation between ICV and fluid intelligence. As for other important confounders, such as head motion and ethnicity, we chose not to include them. Head motion has been shown to explain a relatively low percentage of variance in structural MRI-derived variables in the UK Biobank (Alfaro-Almagro et al., 2021), and poses the risk of introducing additional and unnecessary noise. Furthermore, the sample consists predominantly of participants of white European ancestry (Fry et al., 2017). While ethnicity may act as a potential confounder, the underrepresentation of non-white ethnicities limits the ability to detect ethnicity-related effects.

The two gradient tree boosting models were trained using XGBoost on an 80:10:10 train-validation-test set split. In order to find a good model a hyperparameter search was performed on the training data and its performance evaluated on the validation set. Hyperparameters included in the search were number of trees in the model, learning rate η, L1 and L2-regularization parameters α and λ, maximum depth of regression trees, the fraction of samples used for each regression tree (’subsample’), the fraction of randomly chosen features for creating a regression tree (’colsample_bytree’) and the fraction of randomly sampled features for every split (’colsample_bynode’). Furthermore, the number of early stopping rounds was set to 50 for all models. The hyperparameter search was conducted over a systematic grid, evaluating all possible combinations. Learning rates η ranged from 0.001 and 0.2 in logarithmic steps with additional intermediate values, α values between 0 and 1, logarithmically between $10^{-4}$ and $10^{-1}$ with additional intermediate steps, the same for λ where the logarithmic scales starts at $10^{-3}$. Tree depths for the fluid model were searched in $\{3,4,6,8\}$ and for the age model in $\{4,5,6,7\}$. Additionally, the different sampling parameters ranged between 0.6 and 1, and were partly pre-selected to reduce the number of model configurations.

This broad range of values in the hyperparameter search, particularly for η, α, λ and tree depth enables the exploration of different training strategies and accommodation to different data characteristics and patterns. The final hyperparameter values were chosen based on their performance on the validation set, with preference given to smaller tree depths to reduce complexity if the search yielded multiple comparably well-performing models. Generally, we observed a consistent trend where lower tree depths, i.e., between 3 and 5, were preferred, suggesting that increasing model complexity tended to learn noise rather than signal, and reduced generalizibility. Similarly, learning rates around $10^{-2}$ yielded stable and effective performance, whereas optimal regularization parameters appeared to depend more strongly on the specific combination of other hyperparameters. The final model predicting age consisted of up to 3000 trees with a maximum depth of 5, η=0.02, α=0.1, λ=0.1, ‘subsample’=0.8, ‘colsample_bynode’=0.8, ‘colsample_bytree’=1 and explained 58% of the variance in the test set. The model predicting fluid intelligence was built using up to 1000 trees with a maximum depth of 3, η=0.02, α=0.2, λ = 1, ‘subsample’=0.6, ‘colsample_bynode’=0.6, ‘colsample_bytree’=0.1, and was able to establish a weak connection between the response and the brain measures, explaining 10% of the variance in the training set and 6% in the test set. More details on the performance of each model is given in Table 1. While the gap between training and test performance may suggest overfitting, the validation and test errors are nearly identical, indicating good generalization and no overfitting to the validation set. Some degree of overfitting to the training data is inevitable in supervised learning tasks, as a well-fitted models will capture both signal and noise (Hastie et al., 2009).

**Table 1:** Standard deviation and performance metrics for models age (residualized) and fluid intelligence (not residualized) on training, validation and test data set

	SD	RMSE	MAE	R^2^
Age				
Training	7.48	3.34	2.67	0.80
Validation	7.53	4.93	3.98	0.57
Test	7.50	4.87	3.90	0.58
Fluid Intelligence				
Training	2.06	1.96	1.57	0.10
Validation	2.06	2.00	1.60	0.06
Test	2.04	1.98	1.59	0.06

### Explaining the Model and its Predictions

#### Grouping of Features

Due to computational efficiency and algorithmic requirements, we applied the group Shapley approach (Jullum et al., 2021) when computing conditional Shapley values (Aas et al., 2021). We divided the 179 features into 16 groups, chosen with regards to their theoretical relevance in order to facilitate the interpretation of these groups.

Cortical regions based on the Desikan-Killiany-Tourville atlas were grouped into the frontal, parietal, temporal, and occipital lobes, as well as the limbic cortex, including the insular cortex (Klein & Tourville, 2012). As for subcortical regions, groups consisted of area, mean thickness, and mean intensity for both hemispheres. Furthermore, we created separate groups for the ventricles, the cerebellum, and cerebral white matter. We also used an alternative grouping in which the lobes and the limbic cortex were combined into a single group comprising the cortex, resulting in a total of 12 groups.

All groups and their corresponding brain structures are shown in Table 2.

**Table 2:** Grouping of brain regions into relevant brain structures

Group	Brain Structures
Frontal Lobe	Caudal Middle Frontal Gyrus,
	Lateral Orbitofrontal Cortex,
	Medial Orbitofrontal Cortex,
	Pars Opercularis,
	Pars Orbitalis,
	Pars Triangularis,
	Precentral Gyrus,
	Rostral Middle Frontal Gyrus,
	Superior Frontal Gyrus
Temporal Lobe	Entorhinal Cortex,
	Fusiform Gyrus,
	Inferior Temporal Gyrus,
	Middle Temporal Gyrus,
	Superior Temporal Gyrus,
	Transverse Temporal Gyrus
Occipital Lobe	Cuneus,
	Lateral Occipital Cortex,
	Lingual Gyrus,
	Pericalcarine Cortex
Parietal Lobe	Inferior Parietal Lobule,
	Paracentral Lobule,
	Postcentral Gyrus,
	Superior Parietal Lobule,
	Supramarginal Gyrus
Limbic Cortex	Caudal Anterior Cingulate Cortex,
	Isthmus of the Cingulate Cortex,
	Parahippocampal Gyrus,
	Posterior Cingulate Cortex,
	Rostral Anterior Cingulate Cortex
Ventricle	3rd Ventricle,
	4th Ventricle,
	5th Ventricle,
	Inferior Lateral Ventricle,
	Lateral Ventricle
Corpus Callosum	Anterior Corpus Callosum,
	Central Corpus Callosum,
	Mid Anterior Corpus Callosum,
	Mid Posterior Corpus Callosum,
	Posterior Corpus Callosum
Caudate	Caudate
Hippocampus	Hippocampus
Pallidum	Pallidum
Putamen	Putamen
Thalamus	Thalamus
Amygdala	Amygdala
Accumbens	Accumbens
Cerebellum	Cerebellum Cortex,
	Cerebellum White Matter
Cerebral White Matter	Cerebral White Matter

### Interpretability

Since model performance for problems relating MRI-derived features to age or cognition is generally moderate to low, it is strongly discouraged to interpret individual predictions in isolation. Instead, our goal for local interpretability methods such as KernelSHAP and conditional Shapley values is to extract global patterns by combining a large number of local explanations and ranking features according to their mean absolute contribution values.

As discussed earlier, computing exact Shapley values, be it KernelSHAP values or conditional Shapley values, is very computationally intense, even for a single observation. In practice, Shapley values are usually obtained through approximations that can be tuned by a number of parameters, significantly impacting computation time and performance. The most dominant parameter is the size of the background data set, i.e., the subset of the training set used to estimate the conditional distribution in Eq. 4 or the marginal distribution in Eq. 5 for KernelSHAP. In the case of conditional Shapley values, when the multivariate Gaussian approximation is used, the background data is used to estimate the conditional mean and covariance in Eqs. 6 and 7. When the empirical conditional distribution is used instead, the background data is also used to compute the Mahalanobis distance Eq. 9 and the weight function Eq. 10.

The size of the background data should be chosen such that it can accurately represent the distribution of the entire dataset, which heavily depends on the complexity and dimensionality of the data. In our experiments, we have chosen 1000 randomly sampled observations from the training set as the background data. These samples were the same for both KernelSHAP values and conditional Shapley values.

To obtain global feature importance rankings, we computed KernelSHAP and conditional Shapley contributions for 250 observations from both the training set and the test set, using the same 1000 samples as background to ensure comparability across methods.

To counteract a possible Rashomon effect, we split our dataset into four parts, each containing about 10000 observations, and trained four new models while keeping the model’s hyperparameters the same as in the initial model trained on all data (Fisher et al., 2019). After rerunning all global methods on all models, we obtained four different rankings for each method. The rankings were compared within methods using a weighted version of Kendall’s τ rank correlation (Kendall, 1970). This approach gives an indication of the robustness of the models in identifying the driving features underlying the data. In particular, τ=1 indicates perfect agreement between two rankings, whereas τ=0 indicates that the rankings are completely random. To further assess the sensitivity of the feature importance rankings to reasonable changes to the hyperparameters, we trained additional models with setting the learning rate to half and double the optimal value, as well as tree depth to one level below and above the optimal level, as these hyperparameters appeared to be the most influential. We then evaluated consistency of the Shapley-based feature rankings based on these models on training and test set using Kendall’s τ. While robustness and sensitivity analyses give indication of the stability of feature importance rankings across different models and data splits, it does not ensure that important features represent biologically meaningful or statistically significant signal. Given the modest predictive performance for modelling age, and even lower performance for cognitive traits, interpretability results must be taken with caution. As interpretability methods derive contributions from the model’s predictions rather than the observed outcomes, there is a possibility that contributions reflect noise artifacts rather than true underlying signal. To address this issue, we implemented a validation step in which we assessed whether the contributions of features were statistically associated with the observed outcome in the test set. For each feature $i \in \{1, \dots , m\}$, we tested statistical significance through modelling the outcome *y* as

$$\begin{aligned} y = \beta _0 + f(\varphi _i) + \epsilon , \end{aligned}$$

with $\varphi _i$ denoting the contribution of feature *i*, and f(·) denoting a linear or smooth function estimated through a linear and generalized additive model (GAM), respectively, to capture both linear and non-linear associations. While Shapley-based methods are inherently additive, they use predictions rather than the actual observation for their computations. This is an important distinction as observation and prediction might not even be linearly associated, especially in the context of limited predictive power and complex interactions in the data. The p-values of each feature’s contribution are then FDR-corrected using the Benjamini-Hochberg procedure.

**Fig. 1:**
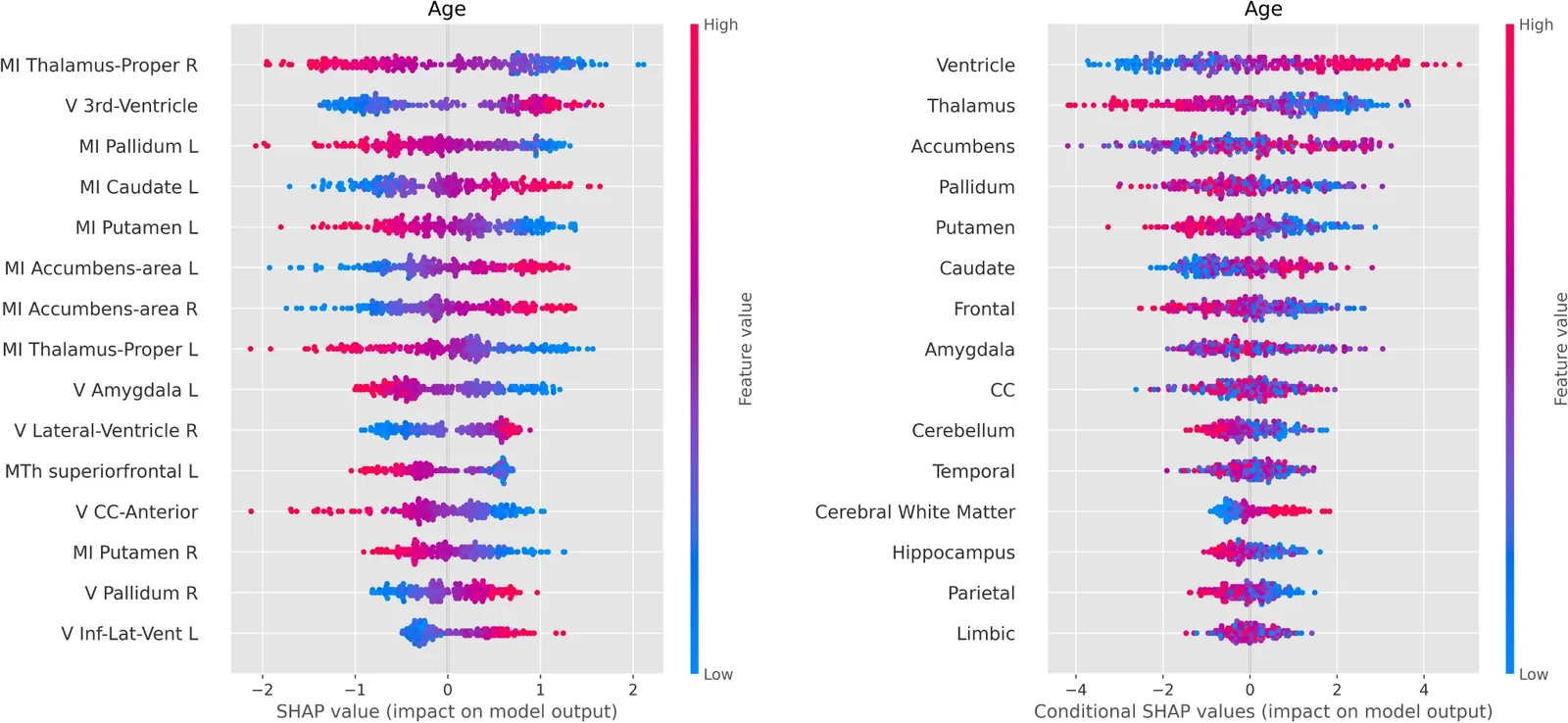
KernelSHAP (left) and conditional Shapley (right) values for the XGBoost model predicting age on 250 observations from the training set. High feature values are colored in red and low feature values in blue. The vertical axis represents the first 15 most important features

Since the computation of the 250 Shapley explanations can be done independently of each other, the often time-costly computation can be accelerated by implementing a straight forward parallelized approach, or by using distributed computing tools such as Apache Spark (Zaharia et al., 2016) in the KernelSHAP method. For SAGE computations, we used only 50 observations for the imputer to optimize computation time, as we did not observe an improvement in Kendall’s τ when computing it with the entire background set consisting of 1000 observations for the age model. As a result, we decided to continue with 50 observations in all further SAGE computations.

### Age

We first present interpretability results for the age prediction model, which achieves higher predictive performance compared to the fluid intelligence model. To better understand the patterns the model has learned during training, we show feature importance of Shapley-based methods on the training set. However, if the distributions of training and test set are statistically identical and proper model training and regularization has been performed, results should be consistent. For plots on the test set, see Supplementary Material. As global versions of Shapley methods rely on the distribution of the computed contribution values and are hence more directly influenced by the data itself than other methods, a well-balanced data set is essential not only for effective model training but also for interpretation using data-driven approaches. Feature importance results of the KernelSHAP and conditional Shapley methods are visualized in Fig. 1. The vertical axis indicates the 15 most important features/feature groups for explaining the prediction in terms of their average magnitude. Each row depicts the distribution of the computed Shapley values, with each point representing a single participant and colors red and blue indicating high and low feature values, respectively. Since corresponding feature values for the grouped conditional Shapley values are not readily available, we created a substitute feature value by determining the quartile each feature belonging to the group corresponds to and averaging over them. It should be noted, however, that, while thickness, area and volume generally have the same directional relationship, i.e., increased thickness, area, and volume indicate lower chronological age, this does not necessarily hold true for intensities. The conditional Shapley values were computed using ShapR and plotted in the framework provided by SHAP.

**Fig. 2:**
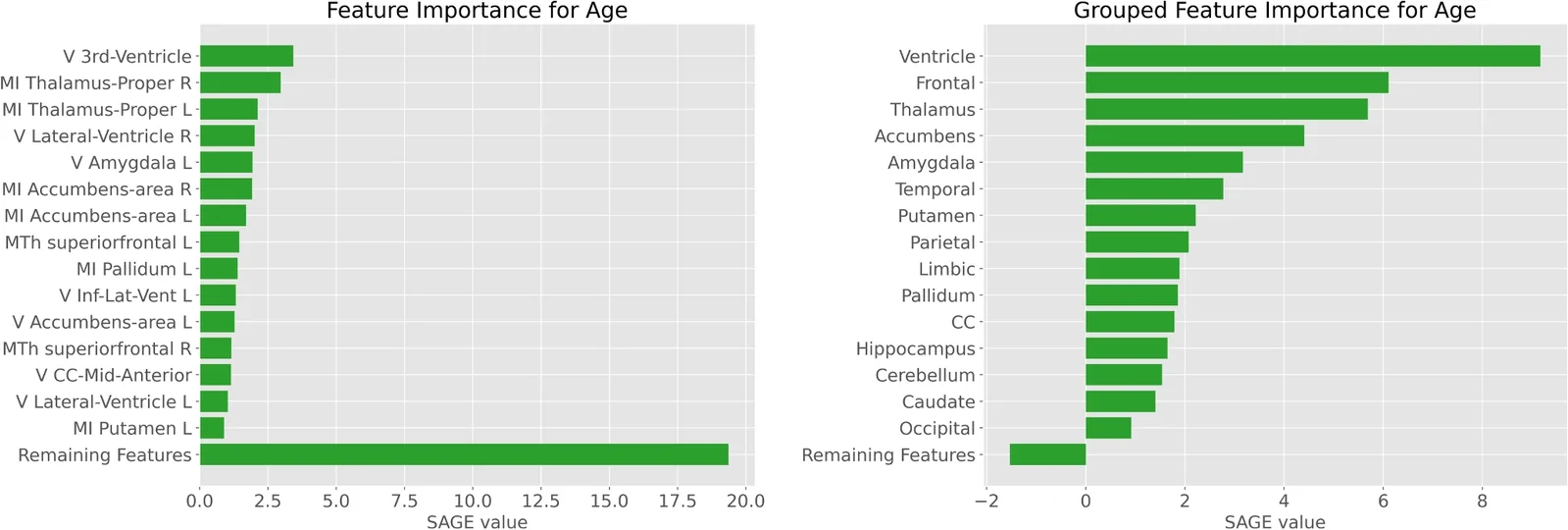
SAGE feature importance with all 179 features (left) and grouped brain structures (right)

The KernelSHAP plot on the left of Fig. 1 shows that 8 of the first 15 most predictive features are mean intensities, specifically of the thalamus (L, R), pallidum (L), caudate nucleus (L), putamen (L, R), nucleus accumbens (L, R) with different directional relationships. For example, higher mean intensity values of the thalamus indicate a lower chronological age, whereas lower mean intensities of the nucleus accumbens indicate a lower chronological age. Furthermore, the KernelSHAP method determined ventricular volumes to be among the features driving the prediction, with lower ventricular volume consistently indicating lower chronological age. The conditional Shapley values identified the combined contribution of the ventricles as the most predictive, followed by contributions of subcortical measures of the thalamus, nucleus accumbens and pallidum. SAGE values, as in Fig. 2, depict the first 15 most important features, with the x-axis indicating the estimated reduction loss attributable to that feature. As seen above in the KernelSHAP plots (Fig. 1), SAGE identifies the ventricles as well as the mean intensities of the thalamus (L, R), and the nucleus accumbens (L, R) as the most predictive features. Furthermore, SAGE allows for grouped feature importance calculations, as shown in the right of Fig. 2. Rankings from the grouped SAGE values and the mean absolute conditional Shapley values display a similar ordering with the ventricles, thalamus and nucleus accumbens far ahead. However, SAGE considers the frontal lobe to be more predictive compared to conditional Shapley values.

Before evaluating the validity of the predictive features with respect to the actual observation, we first assess robustness and sensitivity of the computed feature importance rankings. Specifically, we examine models trained on different subsets of the datasets with fixed hyperparameters and models trained with varying hyperparameters but fixed train and test sets, as described in Section Interpretability.

Considering the large sample size of the UK Biobank data, we expect similar data distributions for the smaller models (n=10000). Surprisingly, the pairwise model comparison within the data-driven approaches, τ within (0.79, 0.81) for KernelSHAP and within (0.95, 0.98) for the conditional Shapley values, was significantly higher than for the SAGE computation; Kendall’s τ for the computation for all features lies within (0.72, 0.77) and within (0.84, 0.92) for the group approach. Nevertheless, it is expected that Kendall’s τ is higher for grouped feature importance rankings.

**Table 3:** Features with significant linear (linear regression) or non-linear (GAM) associations with age in the test after FDR correction for conditional Shapley explanations

Feature	p-value linear	significance	p-value gam	significance
Accumbens	<0.001	***	<0.001	***
Amygdala	<0.001	***	<0.001	***
Caudate	<0.001	***	<0.001	***
CC	<0.001	***	0.002	**
Cerebral White Matter	0.008	**	0.008	**
Frontal	<0.001	***	<0.001	***
Hippocampus	<0.001	***	<0.001	***
Parietal	0.003	**	0.009	**
Thalamus	<0.001	***	<0.001	***
Ventricle	<0.001	***	<0.001	***

Significance levels are indicated as follows: p<0.05 (*), p<0.01 (**), p<0.001 (***)

**Fig. 3:**
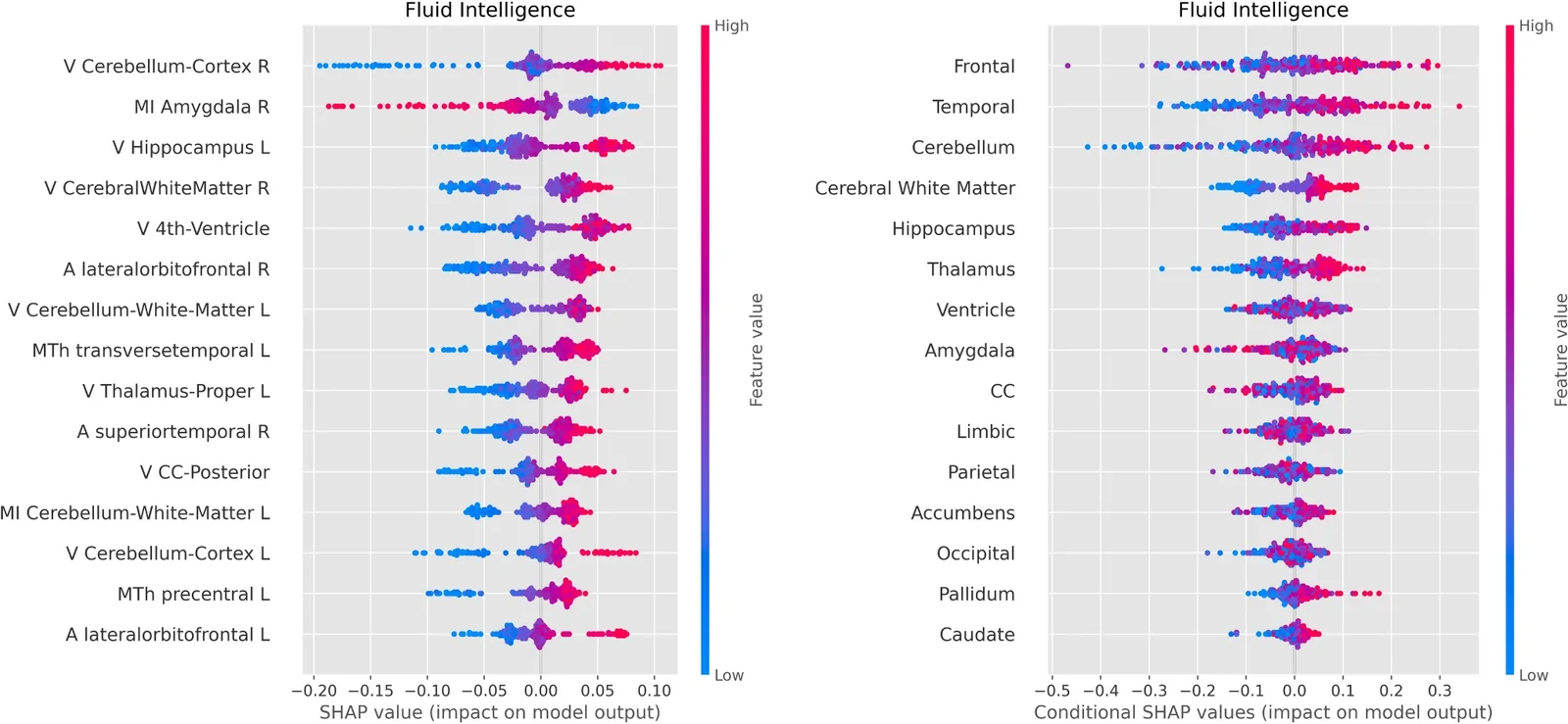
KernelSHAP (left) and conditional Shapley (right) values for the XGBoost model predicting fluid intelligence on 250 observations from the training set

As for the sensitivity of the Shapley values towards changes in model hyperparameters, we observed high consistency in the feature importance rankings across different model configurations and across training and test set. Specifically, Kendall’s τ for KernelSHAP lies within (0.94, 0.98), while for conditional Shapley τ lies within (0.94, 0.99). While conditional Shapley rankings remained consistently highly robust and stable, KernelSHAP showed more variability across data sets and, thus, reduced robustness.

In a next step, we investigated the overlap of the rankings produced by the different models and methods. As rankings were highly consistent across different hyperparameters and therein across training and test set, we restricted this analysis to models trained on different subsets of the training set. The mean intensities of the thalamus (R) and pallidum (L), as well as the volume of the third ventricle, were consistently among the five most predictive features across all models and methods. In addition, the mean intensities of the nucleus accumbens (L, R) and thalamus (L) were among the top 10 predictive features for the interpretation using non-grouped brain measures. For the cortical measures divided into the different lobes, the ventricular volumes were systematically found to be the most predictive of age, followed by the frontal lobe, the nucleus accumbens, and the thalamus. When grouping all cortical measures together, the cortex was less predictive than both the nucleus accumbens and the thalamus. Further details can be found in the Supplementary Materials. Although it is likely that grouped effects consisting of a greater number of features are detected to be more predictive, it is possible that too many weakly predictive features in a group average out the effect of highly predictive features, as in the case of the cortex and the frontal lobe.

Lastly, we evaluated the validity of the features identified as predictive. It is important to stress, that local contribution methods, such as Shapley-based approaches, try to explain the model’s prediction rather than the actual outcome. Purely global methods, such as SAGE, do not allow for instance-level validation tests, whereas KernelSHAP and conditional Shapley enable the assessment of their relevance for the outcome, as described in Section Interpretability. Table 3 shows brain regions whose contributions, as estimated by conditional Shapley values, are significantly associated with age in the test set, such as the amygdala, hippocampus, thalamus, ventricles, and frontal lobe. Similarly, KernelSHAP identifies significant associations with features corresponding to these regions, see Supplementary Table S1. To verify these findings, significance testing was additionally performed across different hyperparameter settings and data splits, as described in Section Interpretability. Most commonly, features identified as significant remained so consistently across different hyperparameters or across different data splits. However, the test set produced fewer significant brain regions compared to the training set. Across all models, the nucleus accumbens, the amygdala, the hippocampus, the thalamus, as well as the frontal lobe and the ventricles were consistently linked to age, see Supplementary Tables S2 and S3. Specifically, the KernelSHAP method highlighted the mean intensities, particularly of the nucleus accumbens (L,R) and the thalamus (L,R), as well as cortical thickness of regions of the frontal and temporal lobes.

**Fig. 4:**
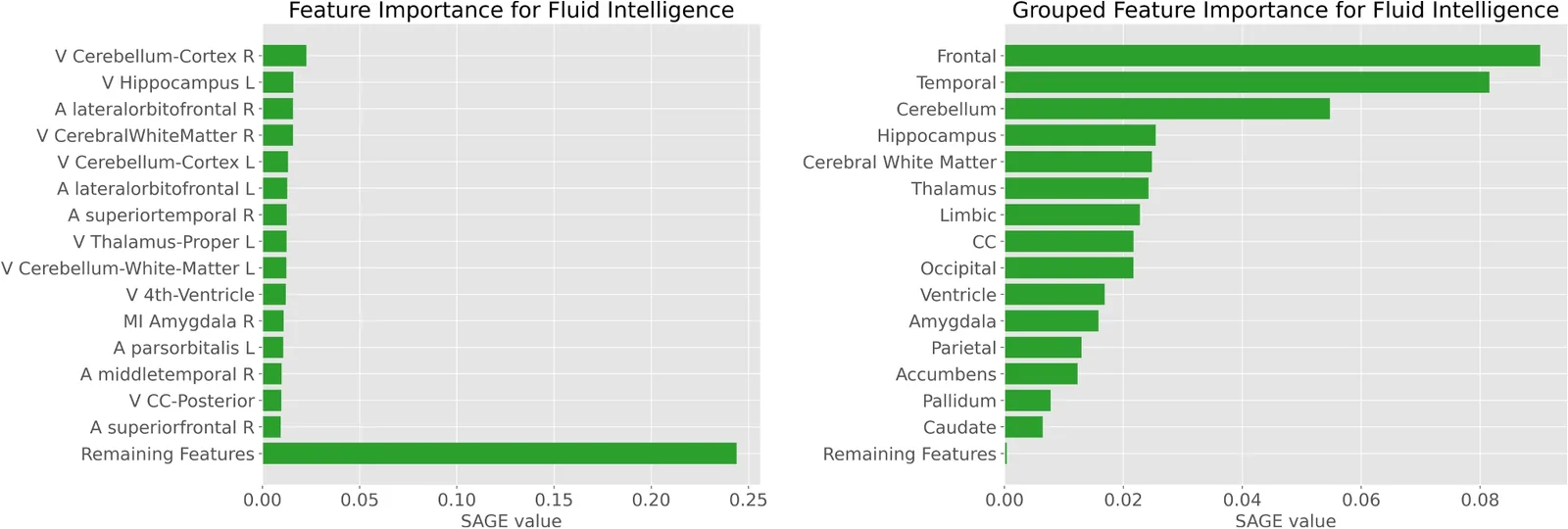
SAGE feature importance with all 179 features (left) and grouped brain structures (right)

### Fluid Intelligence

The same framework used for the age model, as described in Sections Interpretability and Age, was applied, and plots can be interpreted in the same way. The KernelSHAP method shown in Fig. 3 indicates that the volumes of the cerebellar cortex (L,R), cerebral white matter (R), hippocampus (L) and thalamus (L), as well as measures belonging to the frontal and temporal lobes, such as the areas of the lateral orbitofrontal cortex (L, R) and the superior temporal gyrus (R), are predictive of fluid intelligence. Similarly, these measures are estimated by SAGE to be predictive, as shown in Fig. 4. Other regions of the temporal and frontal lobes such as the pars orbitalis, the middle temporal gyrus, and the superior frontal gyrus were found to be driving the prediction of fluid intelligence by SAGE. In general, the contributions are small and at a similar level for most of the brain measures, see in particular Fig. 4. The Kendall’s τ values for the KernelSHAP method lie within (0.20, 0.39), compared to (0.14, 0.36) for the SAGE method using all brain measures. Consistency across different hyperparameters settings in the model was high on training and test set with Kendall’s τ within (0.84, 0.97) for KernelSHAP and (0.82, 0.97) for conditional Shapley values, suggesting that hyperparameters within a reasonable range of the optimal configuration play only a minor role in determining feature contribution patterns. Regarding the grouped feature importance estimation, the conditional Shapley values (Fig. 3) and the grouped SAGE values (Fig. 4) agree that the frontal and the temporal lobes contribute the most to the prediction of fluid intelligence, followed by the cerebellum. The contributions of the hippocampus, cerebral white matter and thalamus are quite a lot smaller, but still substantial. Significantly higher pairwise correlations of feature importance rankings within the grouped approach were observed with Kendall’s τ within (0.81, 0.90) for the conditional Shapley values and (0.71, 0.87) for the grouped SAGE values.

Finally, in terms of validity, Tables 4 and 5 show that the frontal and temporal lobes, and their associated features, are significantly associated with fluid intelligence in the test set, along with subcortical measures of regions such as the thalamus and hippocampus, and cerebral white matter. Additionally, the cerebellum showed consistent associations. Notably, among cortical features, significant associations with the outcome were observed almost exclusively for surface area measures. Furthermore, it is important to note that not all features identified as highly influential for the prediction are significantly associated with the outcome, suggesting that some of the feature contributions may reflect learned noise rather than biological signal.

**Table 4:** Features with significant linear (linear regression) and non-linear (GAM) associations with fluid intelligence on the test set after FDR correction for KernelSHAP explanations

Feature	p-value linear	significance	p-value gam	significance
A caudalanteriorcingulate L	0.054		0.039	*
A cuneus R	0.025	*	0.026	*
A fusiform L	<0.001	***	<0.001	***
A inferiorparietal L	0.814		0.031	*
A inferiortemporal R	0.05	*	0.03	*
A insula R	0.05	*	0.05	*
A lateraloccipital L	0.05	*	0.072	
A lateralorbitofrontal L	0.004	**	0.013	*
A lateralorbitofrontal R	0.05	*	0.048	*
A medialorbitofrontal L	0.015	*	0.029	*
A middletemporal L	0.025	*	0.035	*
A middletemporal R	0.001	**	0.001	**
A paracentral L	0.006	**	0.006	**
A parsopercularis L	0.001	**	0.006	**
A parsorbitalis L	0.001	**	0.001	**
A posteriorcingulate L	0.018	*	0.026	*
A rostralmiddlefrontal R	0.03	*	0.059	
A superiorfrontal L	0.013	*	0.001	**
A superiorfrontal R	0.001	**	0.001	**
A superiortemporal L	0.003	**	0.006	**
A superiortemporal R	<0.001	***	<0.001	***
A transversetemporal L	0.018	*	0.007	**
A transversetemporal R	0.006	**	0.008	**
MTh rostralmiddlefrontal R	0.305		0.019	*
V Accumbens area L	0.008	**	0.008	**
V Amygdala L	0.05	*	0.048	*
V Amygdala R	0.02	*	0.021	*
V Cerebellum Cortex L	0.016	*	0.02	*
V Cerebellum White Matter L	0.033	*	0.065	
V CerebralWhiteMatter L	<0.001	***	<0.001	***
V CerebralWhiteMatter R	<0.001	***	<0.001	***
V Hippocampus L	<0.001	***	0.001	**
V Hippocampus R	0.013	*	0.012	*
V Thalamus Proper L	0.003	**	0.006	**
V Thalamus Proper R	<0.001	***	<0.001	***

**Table 5:** Features with significant linear (linear regression) or non-linear (GAM) associations with fluid intelligence on the test set after FDR correction for conditional Shapley explanations

Feature	p-value linear	significance	p-value gam	significance
Accumbens	0.012	*	0.012	*
CC	0.048	*	0.09	
Cerebellum	0.048	*	0.096	
Cerebral White Matter	<0.001	***	<0.001	***
Frontal	<0.001	***	<0.001	***
Hippocampus	<0.001	***	<0.001	***
Parietal	0.002	**	0.002	**
Temporal	<0.001	***	<0.001	***
Thalamus	0.01	**	0.01	*

## Discussion

In this study, we tried to demonstrate, evaluate and compare multiple local and global interpretability strategies on models predicting age and fluid intelligence trained on the well-curated UK Biobank dataset. The age model achieved strong predictive performance on the test set with an R$^2 = 0.58$, whereas the model predicting fluid intelligence demonstrated limited predictive accuracy, explaining only 6% of the variance in the test set with a correlation between predicted and actual response of r=0.24 (p<0.005). Despite low predictive performance, the interpretability analysis reveals theoretically relevant associations between brain structure and cognition that are consistent with existing literature. As ventricular enlargement is highly associated with age, as is atrophy in most subcortical regions and in the cortex (Walhovd et al., 2005, 2011), the focus for age predictions from tabular structural MRI data commonly lies on morphometry. However, we demonstrated that there are also strong associations between age and intensity measures, which align with findings from previous studies (Salat et al., 2009). In fact, rerunning the model using only unresidualized mean intensity features yields R$^2 = 0.43$ on the test set. Although T1-weighted signal intensities are not direct measures of T1 relaxation times, they are heavily influenced by them, as well as by scanner hardware and acquisition parameters. Nevertheless, mean intensity measures can indirectly reflect biologically meaningful tissue properties relevant to neurobiological ageing, such as changes in myelin content, iron concentration, and tissue water (Stüber et al., 2014). The consistent associations and relatively strong predictive performance indicate that these features may serve as markers of age-related neurobiological changes and represent promising targets for investigating the neurobiological mechanisms reflected in imaging derived measures.

Although there is clear evidence for the predictability of chronological age based on brain measures derived from structural MRI, human intelligence, particularly fluid intelligence, remains poorly understood. While morphometry has been shown to be associated with fluid intelligence, the predictive power of structural MRI for cognitive performance is generally low. Noise in both MRI imaging and neuropsychological test scores further complicates the modelling of accurate relationships. Moreover, it is important to note that the fluid intelligence test used by the UK Biobank is a short and simple test potentially lacking the depth of more comprehensive tests like WAIS (Wechsler, 2012) or Raven’s Matrices (Raven, 2000) for assessing fluid intelligence. An additional challenge is the latent nature of fluid intelligence itself, as it is inferred rather than directly measured. While approaches such as structural equation modelling are better suited to account for measurement error, they are not feasible in settings with high-dimensional data.

As shown by Wang et al. (2024), whether and how intracranial volume (ICV) is accounted for in single effect models impacts the detection of statistically significant associations between fluid intelligence and different brain regions. While residualizing in single effect models has been proven to be efficient, regressing out ICV for all features for the machine learning model for fluid intelligence has inadvertently introduced an ICV effect in some features. This does not necessarily impact model performance, but leads to misidentification of driving brain measures due to the correlation between fluid intelligence and ICV. While residualization was applied to control for sex and ICV effects in the age model, it should be acknowledged that residualizing may introduce biases and may affect and distort underlying patterns, particularly as more covariates are included. Conversely, omitting confounding variables may shift the interpretation of important features, as models may detect shared variance between brain measures and confounds such as age. Confounding control strategies should be chosen carefully, as inclusion and exclusion of confounding variables, as well as residualization with respect to them, each imposes a different interpretative implication in relation to cognitive traits.

In our experiments, we observed indications of a relationship between fluid intelligence and the frontal and temporal lobes (Yuan et al., 2018) and specifically with the area of the right superior temporal gyrus (Liu et al., 2023) amongst others. Furthermore, our findings support previous reports of the involvement of the cerebellum (Anat et al., 2024) and the hippocampus (Reuben et al., 2011) in predicting fluid intelligence.

As for the methodological aspects of the interpretability methods themselves, Shapley values emerge as a robust approach for global interpretability despite their inherently data-driven framework. The choice between the marginal and conditional approach for the Shapley values remains a subject of debate in the field of interpretability, and according to Chen et al. (2020), it ultimately comes down to whether the interpretation should be "true to the model or true to the data". In an optimal setting, being true to the model and being true to the data coincide, and the model accurately maps the underlying relationships. However, this is not necessarily the case for dependent features resulting from a shared latent variable. In such cases, the decision to account for dependencies or not depends on the type of question being addressed through interpretable machine learning.

While it is desirable and necessary to explain the model’s decision in health care to build trust, detect biases in the model, and fulfill regulatory requirements for applications like diagnosing Alzheimer’s disease or strokes in patients, the primary focus in this context lies on developing models for future use in clinical practice. On the other hand, the use of machine learning for knowledge building is becoming increasingly popular. In these instances, the model itself serves as a means to study and interpret associations between input and response. Hence, accounting for dependence structures through conditional Shapley values according to Aas et al. (2021) seems to be the preferred choice.

Nonetheless, Janzing et al. (2019) has strongly discouraged researchers from modifying SHAP values to account for dependence structures, arguing that it *falsely* attributes feature importance to irrelevant features in the model, and has advocated for the incorporation of causal relationships through the Pearl’s do-operator (Pearl, 2009). The expectation in the contribution function Eq. 4 is then taken as the interventional conditional expectation, $\nu (\mathcal {S}) = E(f(x) | \text {do} (\mathcal {S}))$, which coincides with the marginal distribution. The interventional approach implies that it is conceptually, rather than physically, possible to intervene on single features, i.e., single brain regions can be made arbitrarily big or small, independent of each other, and hereby breaking the dependencies allowing for a causal impact of features. This type of intervention is not realistic for brain measures. However, the observational conditional expectation, which coincides with the conditional expectation used in Eq. 4 retains the dependence structure by working with observed data without assuming the ability to intervene on features independently.

Moreover, we observed that although mean absolute Shapley values are observation-dependent, their obtained rankings across the four split datasets showed very high correlations, with the grouped conditional Shapley version naturally exhibiting a higher correlation due to shorter ranking lists. As for their SAGE counterparts, we observed a lower weighted Kendall’s τ.

## Conclusion

Shapley values offer a promising and robust tool for estimating feature contributions in machine learning models, suitable for both single observation explanation and global behavior investigation. Despite the observation-dependent nature of global Shapley value computation as mean absolute feature importance values, they provide consistent rankings of feature importance. The choice between using marginal or conditional Shapley values should agree with the specific interpretative goal. While marginal Shapley values (SHAP) are suited for analyzing model behavior, conditional Shapley values are a better fit for interpreting data with a complex underlying dependence structure. However, the limitation of analyzing a maximum of 30 features may present some challenges for certain types of research questions; in such cases, a carefully curated grouping strategy tailored to the specific research question should be employed.

We want to emphasize again that interpretability in machine learning is a very complex field, and that a universally optimal tool that addresses all interpretability challenges does not exist. The choice of method depends on the data and the interpretation objective. While certain methods may provide consistent and valuable insights in some scenarios, they may be less effective, and at times, misleading in others. Further research is needed to refine and extend existing methods.

## Supplementary Information

Below is the link to the electronic supplementary material.

Supplementary file 1 (pdf 4670 KB)

## Data Availability

The data used in this study was obtained from the UK Biobank (https://www.ukbiobank.ac.uk/). Due to UKB’s restrictions on data-sharing, the data cannot be shared publicly, but simulated datasets as well as R and Python code are available in the OSF archive (https://osf.io/epmgk/).

## Appendix A: Overview of XGBoost

The prediction for a given set of features $x_{i}$ is given by the sum of the predictions from *K* trees,

14 $$\begin{aligned} \hat{y}_{i} = \phi (x_{i}) = \sum _{k=1}^{K} f_{k}(x_{i}) \end{aligned}$$

where $f_{k}(x_{i})$ are regression trees with $T_{k}$ leaves. Each leaf has a corresponding weight, so the total set of weights is a vector $w_{k} \in \mathbb {R}^{T}$. The learning object is the penalized loss function

15 $$\begin{aligned} \mathcal {L}(\phi )= &  \sum _{i=1}^{n} l\left( \hat{y}_{i}, y_{i}\right) + \sum _{k=1}^{K} \big [\gamma T_{k} + (1/2) \alpha \Vert w_{k}\Vert _{1} \nonumber \\ &  + (1/2)\lambda \Vert w_{k}\Vert _{2}\big ]. \end{aligned}$$

In Eq. 15, $\Vert w_{k}\Vert _{1} = \sum _{j=1}^{T_{k}} |w_{kj}|$ is the L1 norm and $\Vert w_{k}\Vert _{2} = \sqrt{\sum _{j=1}^{T_{k}} w_{kj}^{2}}$ is the L2 norm, and α and λ their corresponding regularization parameters, and γ is an additional parameter penalizing the number of leaves. Overall, the first sum in Eq. 15 is the unpenalized loss and the second sum contains three different penalties for model complexity. In addition, XGBoost prevents overfitting with a shrinkage parameter η which scales each new tree added in Eq. 14 and with subsampling of observations (‘subsample’ parameter in the ’XGBoost’ Python package) and features (parameters ‘colsample_bynode’ and ‘colsample_bytree’ in ‘XGBoost’).

## Appendix B: Overview of LIME

One of the first pioneering contributions to the field of XAI is Local Interpretable Model-Agnostic Explanations (LIME) (Ribeiro et al., 2016). As the name suggests LIME tries to explain predictions made by any model-agnostic supervised model through finding a local approximation with a simpler and more interpretable model. However, these explanations require that the input data can be represented in a way that is understood by humans. While tabular data is interpretable by default, for imaging data, using a patch of continuous pixels instead of a single pixel makes much more sense in the context of interpretability. The goal in LIME is to find an explanation model *g* in a family of interpretable models $\mathcal {G}$, e.g., linear models, such that *g* is very faithful to the actual *f* locally around $\textbf{x}$. The explanation for a specific instance $\textbf{x}$ is then found through

$$\begin{aligned} \xi (x) = \underset{g \in \mathcal {G}}{\textrm{argmax}\;} \mathcal {L}(f,g, \pi _{\textbf{x}} + \Omega (g)), \end{aligned}$$

with $\mathcal {L}(f, g, \pi _{\textbf{x}})$ a loss function taking into account the locality of $\textbf{x}$ via similarity kernel $\pi _{\textbf{x}}$, which assigns more weight to observations closer to $\textbf{x}$, and a penalty function Ω penalizing the interpretability of the approximation *g*. While a local linear approximation is a good choice for giving comprehensive interpretations, explanations can become inaccurate if model *f* is highly nonlinear around $\textbf{x}$. Additionally, obtaining a good interpretation model ξ requires sufficient data density around the instance $\textbf{x}$, depending on the dimension of the feature space.
