# Supplementary material for: Gaining Brain Insights by Tapping into the Black Box: Linking Structural MRI Features to Age and Cognition using Shapley-Based Interpretation Methods
Source: Neuroinformatics. 2025 Oct 22;23(4):52. doi: 10.1007/s12021-025-09737-2 (PMC12546294; doi:10.1007/s12021-025-09737-2)
Supplement: Supplementary file 1 — (pdf 4670 KB) [file 12021_2025_9737_MOESM1_ESM.pdf]

Supplementary material to manuscript "Gaining Brain Insights by Tapping into the Black Box: Linking Structural MRI Features to Age and Cognition using Shapley-Based Interpretation Methods"

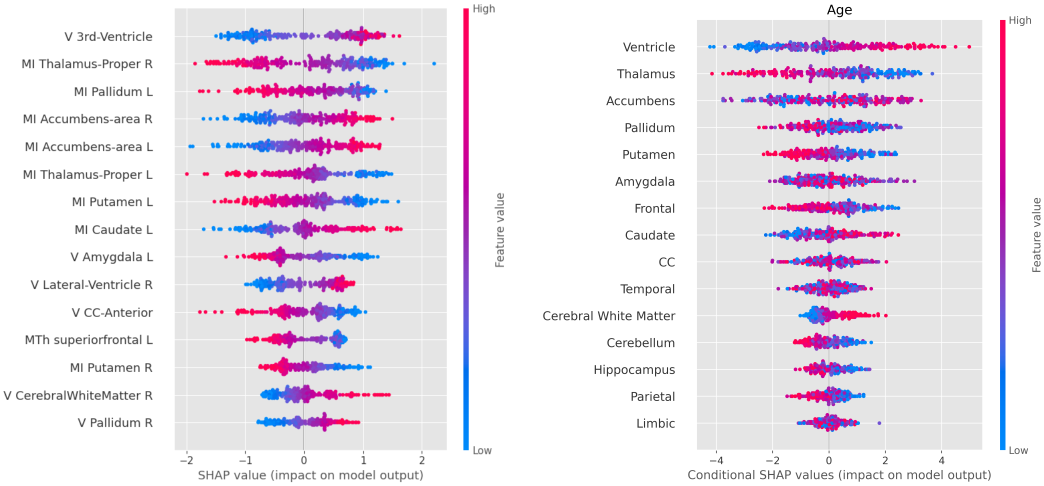

Figure S1: KernelSHAP (left) and conditional Shapley (right) values for the XGBoost model predicting age on 250 observations from the test set.

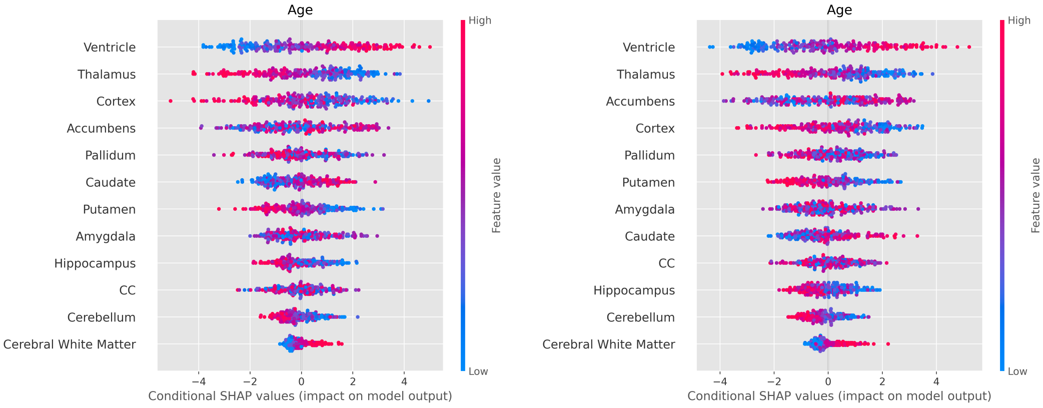

Figure S2: Conditional Shapley values for the XGBoost model predicting age on 250 observations from the training (left) and test (right) set, with all cortical measures grouped together.

Fluid Intelligence

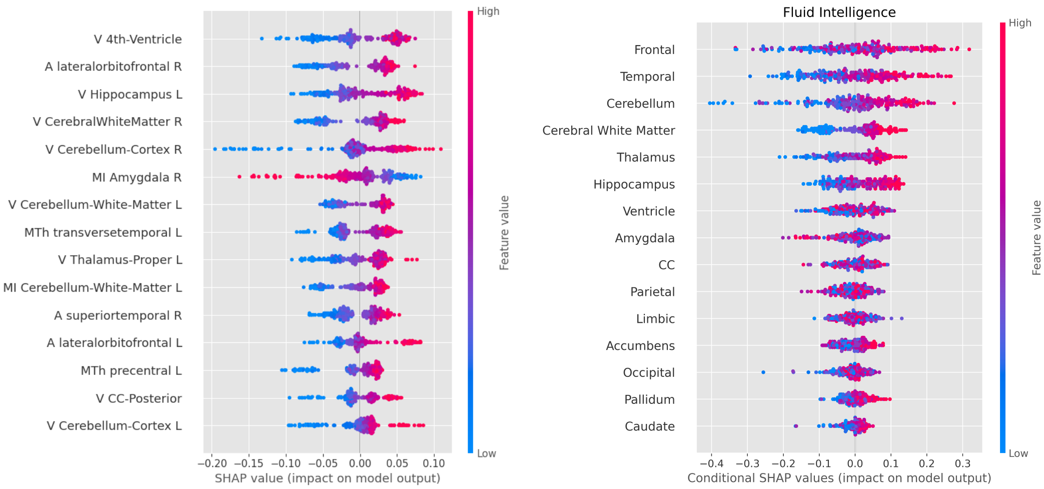

Figure S3: KernelSHAP (left) and conditional Shapley (right) values for the XGBoost model predicting fluid intelligence on 250 observations from the test set.

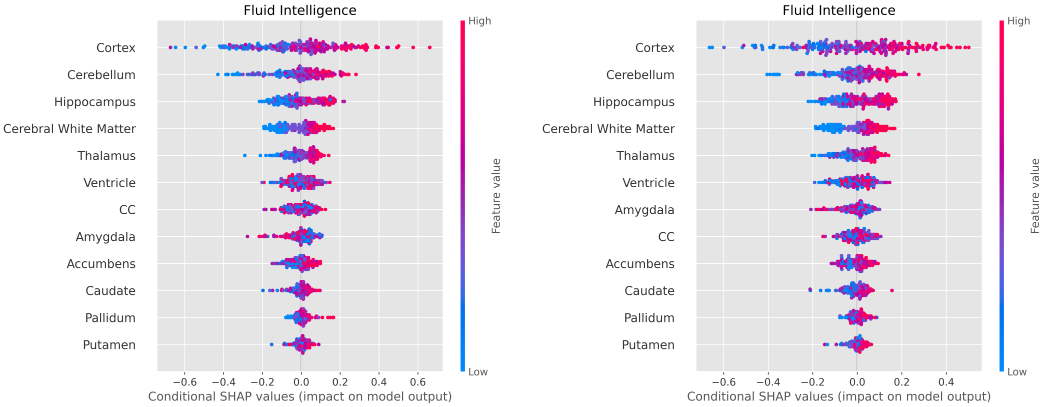

Figure S4: Conditional Shapley values for the XGBoost model predicting fluid intelligence on 250 observations from the training (left) and test (right) set, with all the cortical measures grouped together.

Feature	p-value linear	significance	p-value gam	significance
A lateralorbitofrontal R	0.013	*	0.012	*
A parsorbitalis L	0.158		0.011	*
A parstriangularis R	0.276		0.002	**
A rostralmiddlefrontal R	0.37		0.031	*
MI Accumbens area L	<0.001	***	<0.001	***
MI Accumbens area R	<0.001	***	<0.001	***
MI Amygdala L	0.013	*	0.013	*
MI Amygdala R	0.003	**	0.004	**
MI Caudate L	<0.001	***	0.004	**
MI Caudate R	0.005	**	0.006	**
MI CC Anterior	0.039	*	0.035	*
MI CC Mid Posterior	<0.001	***	<0.001	***
MI CC Posterior	0.01	*	0.016	*
MI Pallidum L	0.005	**	0.011	*

MI Thalamus Proper L	<0.001	***	<0.001	***
MI Thalamus Proper R	<0.001	***	<0.001	***
MTh caudalmiddlefrontal L	0.042	*	0.072	
MTh caudalmiddlefrontal R	<0.001	***	<0.001	***
MTh entorhinal L	0.011	*	0.011	*
MTh entorhinal R	0.022	*	0.018	*
MTh fusiform L	0.011	*	0.006	**
MTh inferiorparietal L	<0.001	***	<0.001	***
MTh inferiorparietal R	<0.001	***	<0.001	***
MTh lateraloccipital L	0.007	**	0.007	**
MTh lateraloccipital R	0.153		0.044	*
MTh lateralorbitofrontal L	0.011	*	0.008	**
MTh lingual L	0.04	*	0.036	*
MTh paracentral L	0.085		0.034	*
MTh paracentral R	0.002	**	0.001	**
MTh parahippocampal L	0.619		0.021	*
MTh parahippocampal R	0.014	*	0.008	**
MTh parsopercularis L	0.003	**	0.008	**
MTh parsorbitalis L	<0.001	***	<0.001	***
MTh parstriangularis L	<0.001	***	<0.001	***
MTh parstriangularis R	<0.001	***	<0.001	***
MTh postcentral L	0.009	**	0.008	**
MTh posteriorcingulate R	0.174		0.016	*
MTh precentral L	0.009	**	0.011	*
MTh precuneus L	<0.001	***	<0.001	***
MTh precuneus R	0.002	**	0.002	**
MTh rostralmiddlefrontal L	<0.001	***	<0.001	***
MTh rostralmiddlefrontal R	0.001	**	0.001	**
MTh superiorfrontal L	<0.001	***	<0.001	***
MTh superiorfrontal R	<0.001	***	<0.001	***
MTh superiorparietal L	0.007	**	0.006	**
MTh superiorparietal R	0.046	*	0.002	**
MTh superiortemporal L	<0.001	***	<0.001	***
MTh superiortemporal R	<0.001	***	<0.001	***
MTh supramarginal R	0.02	*	0.018	*
V 3rd Ventricle	<0.001	***	<0.001	***
V Accumbens area L	<0.001	***	<0.001	***
V Accumbens area R	<0.001	***	<0.001	***
V Amygdala L	<0.001	***	<0.001	***
V CC Anterior	0.025	*	0.023	*
V CC Central	<0.001	***	<0.001	***
V CC Mid Anterior	<0.001	***	<0.001	***
V CC Mid Posterior	<0.001	***	<0.001	***
V Cerebellum White Matter L	0.04	*	0.036	*
V CerebralWhiteMatter L	0.022	*	0.02	*
V CerebralWhiteMatter R	0.037	*	0.033	*
V Hippocampus L	<0.001	***	<0.001	***
V Hippocampus R	<0.001	***	<0.001	***
V Inf Lat Vent L	<0.001	***	<0.001	***
V Inf Lat Vent R	<0.001	***	<0.001	***
V Lateral Ventricle L	<0.001	***	<0.001	***
V Lateral Ventricle R	<0.001	***	<0.001	***
V Putamen L	0.011	*	0.011	*
V Putamen R	0.001	**	<0.001	***
V Thalamus Proper L	<0.001	***	<0.001	***
V Thalamus Proper R	0.004	**	<0.001	***

Table S1: Features with significant linear (linear regression) and non-linear (GAM) associations with age after FDR correction for KernelSHAP explanations.

Table S2: Features significantly associated with age in the training and test sets, either linearly or non-linearly, across the 4 hyperparamter models. A $\times$ indicates significance across all models, a $\sim$ significance in at least one model, and a blank cell no significant association in any model.

Variable	train	test
A rostralmiddlefrontal R	$\times$	$\times$
MI Accumbens area L	$\times$	$\times$
MI Accumbens area R	$\times$	$\times$
MI Amygdala R	$\times$	$\times$
MI CC Mid Posterior	$\times$	$\times$
MI CC Posterior	$\times$	$\times$
MI Pallidum L	$\times$	$\times$
MI Thalamus Proper L	$\times$	$\times$
MI Thalamus Proper R	$\times$	$\times$
MTh caudalmiddlefrontal R	$\times$	$\times$
MTh inferiorparietal L	$\times$	$\times$
MTh inferiorparietal R	$\times$	$\times$
MTh paracentral R	$\times$	$\times$
MTh parsorbitalis L	$\times$	$\times$
MTh parstriangularis L	$\times$	$\times$
MTh parstriangularis R	$\times$	$\times$
MTh precuneus L	$\times$	$\times$
MTh precuneus R	$\times$	$\times$
MTh rostralmiddlefrontal L	$\times$	$\times$
MTh rostralmiddlefrontal R	$\times$	$\times$
MTh superiorfrontal L	$\times$	$\times$
MTh superiorfrontal R	$\times$	$\times$
MTh superiortemporal L	$\times$	$\times$
MTh superiortemporal R	$\times$	$\times$
MTh supramarginal R	$\times$	$\times$
V 3rd Ventricle	$\times$	$\times$
V Accumbens area L	$\times$	$\times$
V Amygdala L	$\times$	$\times$
V CC Anterior	$\times$	$\times$
V CC Central	$\times$	$\times$
V CC Mid Anterior	$\times$	$\times$
V CC Mid Posterior	$\times$	$\times$
V CerebralWhiteMatter L	$\times$	$\times$
V CerebralWhiteMatter R	$\times$	$\times$
V Hippocampus L	$\times$	$\times$
V Hippocampus R	$\times$	$\times$
V Inf Lat Vent L	$\times$	$\times$
V Inf Lat Vent R	$\times$	$\times$
V Lateral Ventricle L	$\times$	$\times$
V Lateral Ventricle R	$\times$	$\times$
V Putamen R	$\times$	$\times$
V Thalamus Proper L	$\times$	$\times$
V Thalamus Proper R	$\times$	$\times$
MI CC Anterior	$\sim$	$\times$
MTh lateraloccipital L	$\sim$	$\times$
MI Amygdala L	$\sim$	$\times$
V Cerebellum White Matter L	$\sim$	$\times$
MI Caudate L		$\times$
MI Caudate R		$\times$
MTh entorhinal R		$\times$
MTh fusiform L		$\times$
MTh lateralorbitofrontal L		$\times$
MTh parsopercularis L		$\times$
A inferiortemporal L	$\times$	$\sim$
A parsorbitalis L	$\times$	$\sim$
A parstriangularis R	$\times$	$\sim$
MI Hippocampus R	$\times$	$\sim$
MTh isthmuscingulate L	$\times$	$\sim$
MTh middletemporal R	$\times$	$\sim$
MTh paracentral L	$\times$	$\sim$
MTh parahippocampal R	$\times$	$\sim$
MTh postcentral R	$\times$	$\sim$
MTh precentral L	$\times$	$\sim$
MTh superiorparietal R	$\times$	$\sim$
A posteriorcingulate R	$\sim$	$\sim$
A rostralmiddlefrontal L	$\sim$	$\sim$
MTh caudalmiddlefrontal L	$\sim$	$\sim$
MTh insula L	$\sim$	$\sim$
MTh precentral R	$\sim$	$\sim$
V Accumbens area R	$\sim$	$\sim$
V Amygdala R	$\sim$	$\sim$
A lateralorbitofrontal R	$\sim$	$\sim$
MTh lateraloccipital R	$\sim$	$\sim$
MTh superiorparietal L	$\sim$	$\sim$
MTh entorhinal L		$\sim$
MTh posteriorcingulate R		$\sim$
A inferiortemporal R	$\times$	
A insula L	$\times$	
A lateralorbitofrontal L	$\times$	
A middletemporal L	$\times$	
A middletemporal R	$\times$	
A parsopercularis L	$\times$	
A parstriangularis L	$\times$	
A precentral L	$\times$	
A precuneus R	$\times$	
A rostralanteriorcingulate L	$\times$	
A superiorparietal R	$\times$	
A supramarginal L	$\times$	
A transversetemporal R	$\times$	
MI Cerebellum Cortex L	$\times$	
MI Cerebellum Cortex R	$\times$	
MI Pallidum R	$\times$	
MI Putamen L	$\times$	
MTh fusiform R	$\times$	
MTh parsorbitalis R	$\times$	
MTh postcentral L	$\times$	
MTh transversetemporal L	$\times$	
V Cerebellum Cortex R	$\times$	
A parahippocampal L	$\sim$	
A parsorbitalis R	$\sim$	
A precuneus L	$\sim$	
MTh inferiortemporal R	$\sim$	
V CC Posterior	$\sim$	
V Cerebellum White Matter R	$\sim$	

Table S3: Feature contributions derived from dependence-aware Shapley values significantly associated with age in the training and test sets, either linearly or non-linearly, across the 4 hyperparameter models. A $\times$ indicates significance across all models, a $\sim$ significance in at least one model, and a blank cell no significant association in any model. Table for KernelSHAP can be found in the Supplementary.

Variable	train	test
Accumbens	$\times$	$\times$
Amygdala	$\times$	$\times$
Caudate	$\times$	$\times$
CC	$\times$	$\times$
Cerebral White Matter	$\times$	$\times$
Frontal	$\times$	$\times$
Hippocampus	$\times$	$\times$
Parietal	$\times$	$\times$
Thalamus	$\times$	$\times$
Ventricle	$\times$	$\times$
Pallidum	$\times$	$\sim$
Limbic	$\times$	
Putamen	$\times$	
Temporal	$\times$	
Cerebellum	$\sim$	

Table S4: Features whose contributions to model predictions show a statistically significant association with fluid intelligence in the training or test set, either linear or non-linear, across 4 hyperparameter models. A × indicates significance across all models, a ∼ denotes significance in at least one model, and a blank cell indicates no significant association in any model. Significant feature contributions derived from KernelSHAP are presented on the left, while dependence-aware contributions are shown on the right.

Feature	train	test
A middletemporal R	×	×
V Cerebellum Cortex L	×	×
V Cerebellum Cortex R	×	×
A superiorfrontal L	∼	×
A superiortemporal L	∼	×
A superiortemporal R	∼	×
A transversetemporal R	∼	×
V CerebralWhiteMatter L	∼	×
A middletemporal L	∼	×
A parsorbitalis L	∼	×
A superiorfrontal R	∼	×
A transversetemporal L	∼	×
V CerebralWhiteMatter R	∼	×
A fusiform L		×
A insula R		×
A lateralorbitofrontal L		×
A paracentral L		×
A parsopercularis L		×
A posteriorcingulate R		×
V Accumbens area L		×
V Cerebellum White Matter L		×
V Hippocampus L		×
V Hippocampus R		×
V Thalamus Proper L		×
V Thalamus Proper R		×
A medialorbitofrontal R	∼	∼
A inferiortemporal R	∼	∼
A lateralorbitofrontal R	∼	∼
A inferiortemporal L		∼
A rostralmiddlefrontal R		∼
A cuneus R		∼
V Amygdala L		∼
V Amygdala R		∼
A caudalmiddlefrontal R	∼	

Feature	train	test
Cerebral White Matter	×	×
Frontal	×	×
Temporal	×	×
Accumbens	∼	×
Hippocampus		×
Parietal		×
Thalamus		×
Cerebellum	×	
Limbic	×	
Pallidum	∼	

Number of top features	Brain measures
**3**	MI Thalamus-Proper R, V 3rd-Ventricle
**5**	MI Accumbens-area R
**7**	MI Accumbens-area L, MI Thalamus Proper L
**10**	V Lateral-Ventricle R, V Amygdala L
**15**	V Inf-Lat-Vent L, MI Pallidum L, MTh superiorfrontal L, V Accumbens-area L
**20**	V Lateral-Ventricle L, MI CC-Mid-Posterior, V Inf-Lat-Vent R
**25**	V CC-Anterior, MI Putamen L
**30**	V Putamen R, MI Pallidum R, MTh superiorfrontal R
**40**	V Hippocampus L, V Thalamus-Proper L, MI Caudate L, V Cerebellum-Cortex R, MTh rostralmiddlefrontal R
**50**	MI Amygdala R, MI Putamen R

Table S5: Cumulative list of the most important features for predicting age identified on all models and through all methods. With increasing number of top features, new brain regions are added to the list.

Nr of top features	Brain measures
**1**	Ventricle
**3**	Frontal
**4**	Accumbens, Thalamus
**7**	Amygdala
**8**	Pallidum
**9**	Parietal, Pallidum
**13**	Limbic, CC, Putamen, Cerebellum

Table S6: Cumulative list of the most important features for predicting age identified on all models and through all methods.

Nr of top features	Brain measures
**1**	Ventricle
**3**	Accumbens, Thalamus
**6**	CC
**7**	Cortex, Amygdala
**8**	Pallidum

Table S7: Cumulative list of the most important features for predicting age identified on all models and through all methods.

**Nr of top features**	**Brain measures**
**1**	Frontal
**3**	Temporal
**5**	Ventricle, Limbic
**6**	Parietal
**8**	CC, Occipital, Cerebellum
**13**	Amygdala, Hippocampus, Thalamus

Table S8: Cumulative list of the most important features for predicting fluid identified on all models and through all methods.

**Nr of top features**	**Brain measures**
**15**	A superiortemporal R
**25**	V Cerebellum-Cortex R
**30**	V CerebralWhiteMatter L, V Thalamus-Proper L, V Thalamus-Proper R,
**50**	A lateralorbitofrontal L, MTh supramarginal R, V 4th-Ventricle, V CC-Posterior

Table S9: Cumulative list of the most important features for predicting age identified on all models and through all methods. With increasing number of top features, new brain regions are added to the list.

**Nr of top features**	**Brain measures**
**2**	Frontal, Temporal
**4**	Limbic
**6**	Cerebellum, Parietal
**8**	Occipital
**9**	Thalamus, CC
**10**	Hippocampus
**13**	Ventricle, Amygdala, Cerebral White Matter

Table S10: Cumulative list of the most important features for predicting fluid identified on all models and through all methods.
